# Effect of Cytotoxic T-Lymphocyte Antigen-4 on the Efficacy of the Fatty Acid-Binding Protein Vaccine Against *Schistosoma japonicum*

**DOI:** 10.3389/fimmu.2019.01022

**Published:** 2019-05-07

**Authors:** Chun-lian Tang, Qun Pan, Ya-ping Xie, Ying Xiong, Rong-hui Zhang, Jin Huang

**Affiliations:** ^1^Wuchang Hospital, Affiliated to Wuhan University of Science and Technology, Wuhan, China; ^2^Department of Clinical Laboratory, Wuhan Fourth Hospital, Puai Hospital, Tongji Medical College, Huazhong University of Science and Technology, Wuhan, China

**Keywords:** *Schistosoma japonicum*, fatty acid-binding protein, regulatory T cells, cytotoxic T-lymphocyte antigen-4, granuloma

## Abstract

The present study evaluated the impact of blocking cytotoxic T-lymphocyte antigen-4 (CTLA-4) activity on the protective effect elicited by the fatty acid binding protein (FABP) vaccine against *Schistosoma japonicum* infection. Mice were randomly divided into uninfected, infected control, anti-CTLA-4 monoclonal antibody (anti-CTLA-4 mAb), FABP, and combination (anti-CTLA-4 mAb and FABP) groups. An assessment of the *S. japonicum* worm and egg burden in the infected mice revealed that the worm reduction-rate induced by FABP administration was increased from 26.58 to 54.61% by co-administration of the monoclonal anti-CTLA antibody (anti-CTLA-4 mAb). Furthermore, the regulatory T cell (Treg) percentage was significantly increased in mice after administration of the anti-CTLA-4 mAb, but not the FABP vaccine, and elevated levels of the cytokines interferon (IFN)-γ, interleukin (IL)-2, IL-4, and IL-5 were observed in infected mice that were administered the anti-CTLA-4 mAb. Notably, the diameter of egg granulomas in the anti-CTLA-4 mAb and combination groups was significantly increased compared to that observed in the infected control group. Together, these results suggest that co-administering the FABP vaccine and anti-CTLA-4 treatment may have synergistically increased the immunoprotective effect of the FABP vaccine by promoting T-helper 1-type immune responses, while incurring increased tissue damage.

## Introduction

Schistosomiasis is a fatal disease that is caused by *Schistosoma* trematode infections, which incurs negative health and economic effects. *Schistosoma* eggs represent the most important pathogenic factor in schistosomiasis, because they incur late-stage symptoms that are difficult to resolve, such as liver fibrosis caused by egg deposition in the host liver (1). Schistosomiasis is generally treated with, but cannot be prevented via the administration of praziquantel; thus, a novel and effective vaccine is needed to improve schistosomiasis control. *Schistosoma* trematodes cannot resynthesize long-chain fatty acids and cholesterol molecules. Accordingly, previous studies have shown that the FABP, which absorbs, transports, and transforms fatty acids in host cell membranes (2), comprises a single effective vaccine against at least two parasites, *Fasciola hepatica* and *S. mansoni* (3). Furthermore, the FABP derived from *F. hepatica* has been reported to exert a strong protective effect against *S. mansoni* infection (4), and similarly, a recombinant *S. mansoni* FABP-pcDNAI plasmid was previously reported to elicit a high level of *sm*FABP-IgG antibody production, and to cause a strong (74.2%) decrease in the number of adult worms, as well as the number of eggs and granulomas (5). Moreover, the specific immune response elicited by, and tolerance of male volunteers (in non-schistosomiasis endemic areas) for a *Sm*FABP vaccine (formulated with a glucopyranosyl lipid A adjuvant, in an oil-in-water emulsion) has been successfully analyzed via landmark, open-knowledge phase-I and IIa clinical trials (6), and is currently being evaluated by phase-II/III trials (7). While the vaccine's mechanism of action remains unclear, it is speculated to be mediated by regulatory T cells (Tregs). Previous research has confirmed that the inadequate immune protection provided by the (gluthatione-S-transferase) GST vaccine may be the result of vaccine-induced Treg production; thus, the anti-CD25 monoclonal antibody (mAb) and cimetidine have been shown to improve the efficacy of the GST vaccine by blocking and reducing the percentage of Tregs, respectively (8, 9).

Tregs comprise various T-cell subsets with inhibitory properties, and have been shown to express phenotypic markers including interleukin (IL)-2-receptor alpha chains, cytotoxic T-lymphocyte-associated protein 4 (CTLA-4), glucocorticoid-induced tumor necrosis factor receptor (GITR), forkhead box protein 3 (Foxp3), and lymphocyte activation gene 3 (LAG3) (10). CTLA-4 belongs to the immunoglobulin-related family of receptors that mediate various aspects of T-cell immune regulation (11). The most important function of CTLA-4 is to inhibit T-cell activation; thus, mice with CTLA-4 gene deficiency exhibit lymphoproliferation and multiple organ lymphocyte infiltration, and die 3–4 weeks after birth (12). Several studies have highlighted the importance of, and protective effect provided by CTLA-4 blockade in vaccine design, and furthermore, have demonstrated that vaccine-induced protective immunity can be enhanced by co-stimulation strategies that reduce the threshold for T-cell activation and/or block immune inhibitors (13). For example, combination immunotherapy in which the vaccine is administered with the anti-CTLA-4 mAb has been demonstrated to significantly enhance anti-tumor immune responses compared to administration of either the vaccine or mAb alone (14). In fact, CTLA-4-binding antibodies have been suggested to have great potential for treating chronic infections, especially when combined with therapeutic vaccines (15). Since the effect of CTLA-4 blockade on the efficacy of FABP vaccination against *S. japonicum* has not been previously reported, the present study assessed the effect (and underlying mechanisms) of the anti-CTLA-4 mAb on the immunoprotective effect of the FABP vaccine in mice infected with *S. japonicum*.

## Materials and Methods

### Animals and Parasites

Forty female BALB/c mice (aged 6–8 weeks) were obtained from the Hubei Province Center for Disease Control and Prevention (China), and randomly divided into five groups (*n* = 8/group), comprising an uninfected, infected control, anti-CTLA-4 mAb, FABP, and combination (FABP and anti-CTLA-4 mAb) group. *Oncomelania hupensis* snails infected with *S. japonicum* were obtained from the Jiangsu Province Institute of Parasitosis Control and Prevention (China), and exposed to light to stimulate cercariae shedding. All experiments in the present study were conducted with the approval of the Animal Research Committee of Wuchang Hospital (No. 2017-0027).

### Experimental Schedule

The mice in the FABP and combination groups were immunized with 100 μg of the FABP vaccine against *S. japonicum* [which recombinant protein was prepared by our laboratory (16)], while those in the other groups were instead administered an equal volume of PBS. Identical “booster” immunizations were administered twice within a 2-week interval. After a further 2 weeks, the mice in the infected (i.e., infected control, anti-CTLA-4 mAb, FABP, and combined) groups were percutaneously infected with 40 ± 1 *S. japonicum* cercariae. Mice in the anti-CTLA-4 mAb and combination groups were intraperitoneally injected with 300 μg of anti-CTLA-4 mAb at 2 weeks post-infection, while those in the other groups were administered an equal volume of PBS. Detailed procedures are shown in Table 1. All mice were sacrificed 5 weeks after infection (i.e., 3 weeks post-anti-CTLA-4 mAb administration).

**Table 1:** Processing of mice in each group.

**Treatment group**	**Number of mice**	**Immunization**	**Parasite challenge**	**Anti-CTLA-4 mAb**	**Number of experiment repeats**
Uninfected	8	PBS as control	No infection	PBS as control	3
Infected-control	8	PBS as control	Operation 2	PBS as control	3
Anti-CTLA-4 mAb	8	PBS as control	Operation 2	Operation 3	3
FABP	8	Operation 1	Operation 2	PBS as control	3
Combination	8	Operation 1	Operation 2	Operation 3	3

*CTLA-4, cytotoxic T-lymphocyte-associated protein 4; FABP, fatty acid-binding protein; mAb, monoclonal antibody; PBS, phosphate-buffered saline; Operation 1, immunization with 100 μg of SjFABP, followed by identical “booster” immunizations that were administered twice within a 2-week interval; Operation 2, 2 weeks after the last immunization, mice were percutaneously infected with 40 ± 1 S. japonicum cercariae; Operation 3, mice were intraperitoneally injected with 300 μg of anti-CTLA-4 mAb at 2 weeks after the initiation of the experiment*.

### Worm- and Egg-Burden Assessment

The portal-vein infusion method previously described by Li et al. (17) was used to retrieve adult worms from the mice in the four infected groups after sacrifice. Briefly, the abdominal and thoracic cavities of the mice were dissected. The mice then underwent left-ventricular perfusion with 20–30 ml/mouse of normal saline (until the mesenteric vessels turned white). Impact force of liquid perfusion caused the worms to be washed out, so that they could be gently clasped with small tweezers and placed into a saline solution. The worms were then counted using an anatomical microscope, and the worm-reduction rate was calculated. Similarly, livers were removed from the mice in the infected groups after sacrifice, and digested with 5% potassium hydroxide. The number of eggs in an 1-ml aliquot of each digested sample was then counted, and the liver egg-reduction rate were calculated according to the method described by Cheng et al. (18). All experiments were performed in triplicate. The remaining liver samples were used for a histopathological granuloma assessment.

### Flow Cytometric Analysis

To evaluate regulatory the Treg percentage, single-cell suspensions of splenocytes were prepared according to the method described by Mo et al. (19). Briefly, resected spleens were ground and filtered through a 200-mesh filter cloth. Red blood cells in the generated cell pellet were cleaved using an erythrocyte lysate (Beyotime, China), and 5 × 10^6^ cells were suspended in Roswell Park Memorial Institute (RPMI)-1640 medium. The cells were then stained with conjugated antibodies including FITC-conjugated anti-mouse CD4, APC-conjugated anti-mouse CD25, and PE-conjugated anti-mouse Foxp3, using a mouse Treg-staining Kit (eBioscience). Finally, they were analyzed using a BD Biosciences FACSCalibur flow cytometer (Becton Dickinson, Mountain View, CA).

### Assessment of Murine Cytokines in Splenic Cell Culture Supernatants

Single-cell suspensions were prepared as above, and 5 × 10^6^ cells retrieved from each spleen suspension were transferred onto 24-well culture plates (Corning, USA). The cell cultures were then incubated (37°C, 5% CO_2_) in RPMI-1640 medium with or without 5 μg/ml Concanavalin (Sigma). After 72 h, the cell cultures were centrifuged, and the resulting supernatants were subjected to an enzyme-linked immunosorbent assay (ELISA) using a commercial kit (eBioscience) to assess cytokine (IL-2, interferon (IFN)-γ, IL-5, and IL-4) levels.

### Histopathological Evaluations

The right hepatic lobe from each mouse in the four infected groups was fixed in 10% formalin, and embedded in a paraffin block. Sections (5-μm thick) were stained with hematoxylin and eosin (H & E), and analyzed using a compound microscope (magnification, × 200). To evaluate histopathological changes among groups, the diameter of the liver granulomas (*n* ≥ 20 granulomas/liver) was measured using IPP 6.0 software (Media Cybemetics, USA).

### Statistical Analysis

All data were analyzed using SPSS 23.0 software, and expressed as the mean ± standard deviation (S.D). An analysis of variance was used to make comparisons between different groups. A *P*-value < 0.05 was considered to indicate statistical significance.

## Results

### Effect of the Anti-CTLA-4 mAb on the Protective Efficacy of the FABP Vaccine Against Murine *S. japonicum* Infection

To investigate the protective effect of the FABP vaccine, the worm and egg burdens exhibited by the eight mice in each group were detected at 5 weeks after infection. The results of this analysis demonstrated that the worm and egg burdens displayed by the FABP and anti-CTLA-4 mAb groups were significantly reduced compared to those exhibited by mice in the infected control group. Moreover, this reduction was greater in the mice treated with both the FABP vaccine and the anti-CTLA-4 mAb than in those that the FABP vaccine was administered alone (Table 2).

**Table 2:** The effect of blocking cytotoxic T-lymphocyte antigen-4 (CTLA-4) activity on the efficacy of the fatty acid-binding protein (FABP) vaccine against murine *S. japonicum* infection.

**Treatment group**	**Worm burden**	**Worm-reduction rate (%)**	**Average number of eggs/1 g liver tissue (× 10^**3**^)**	**Liver egg-reduction rate (%)**
Infected-control	27.95 ± 2.82	Not applicable	23.42 ± 7.91	Not applicable
Anti-CTLA-4 mAb	21.87 ± 3.53	21.75^*^^#^	19.0 ± 5.36	18.89^*^^#^
FABP	20.52 ± 4.62	26.58^*^^#^	16.10 ± 6.58	31.24^*^^#^
Combination	12.69 ± 1.46	54.61^*^	9.54 ± 5.67	59.25^*^

*The worm burden was evaluated via the portal-vein infusion method, and the worm-reduction rate was calculated. Livers were removed from the mice in the infected groups after sacrifice, and digested with 5% potassium hydroxide. The number of eggs in an 1-ml aliquot of each digested sample was then counted, and the liver egg-reduction rate were calculated. Data are presented as the mean ± S.D from three independent combined experiments (n = 8/group)*.

* *P < 0.05 compared to the infected-control group*.

# *P < 0.05 compared to the combination group*.

*Anti-CTLA-4 mAb, anti-CTLA-4 monoclonal antibody*.

### Effect of the Anti-CTLA-4 mAb on Treg Percentage by Splenocytes

**Figure 1:**
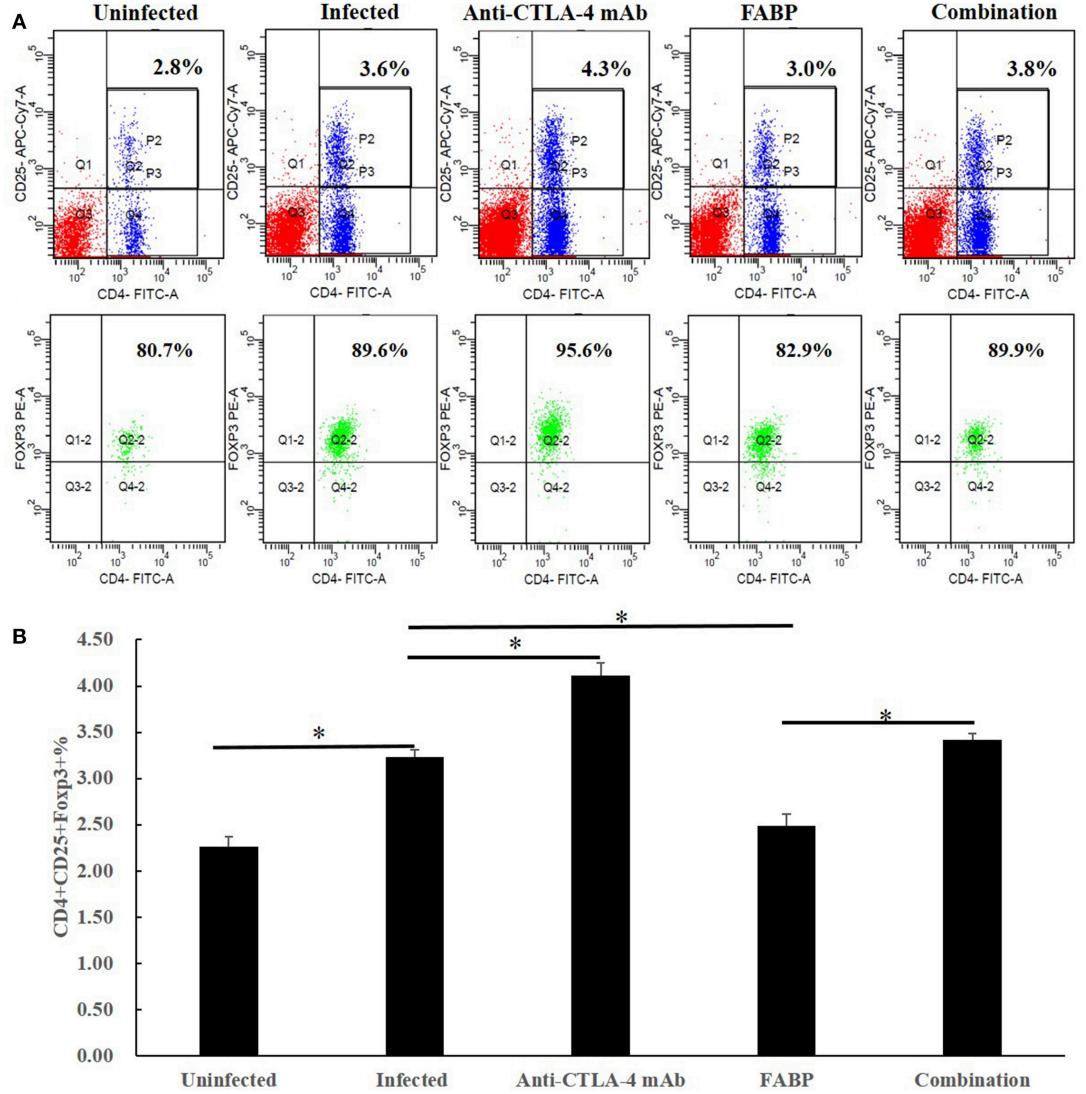
The effect of administering the fatty acid-binding protein (FABP) with and without the anti-cytotoxic T-lymphocyte antigen-4 (CTLA-4) monoclonal antibody (mAb) on the percentage of regulatory T cells (Tregs) in mice infected with *Schistosoma japonicum*. **(A)** Representative flow cytometry data showing splenocyte Treg percentages in mice in the uninfected, infected control, anti-CTLA-4 mAb, FABP, and combination groups at 5 weeks after *S. japonicum* infection. Upper panels: P2 gate shows the frequency of CD4^+^CD25^+^ T cells in isolated splenocytes. Lower panels: Q2–2 gate indicates the proportion of Foxp3^+^ lymphocytes in the P2 gate (upper panels). **(B)** Frequencies of Tregs in total splenocytes in the five mouse treatment groups at 5 weeks post-infection (i.e., 3 weeks post anti-CTLA-4 mAb administration). Data are presented as the mean ± S.D for experiments performed in triplicate. *P < 0.05.

Tregs have been shown to express Foxp3 (20); thus, we herein considered T cells expressing CD4, CD25, and Foxp3 (CD4^+^CD25^+^Foxp3^+^) to be Tregs. As shown in Figure 1, the Treg percentage was increased in splenocytes after *S. japonicum* infection. In the infected background, compared with those of the infected group, the percentages of Tregs in the FABP-treated group were significantly lower, whereas they were significantly higher in the anti-CTLA-4 mAb-treated group (*P* < 0.05). The frequency of Tregs in the combination group was also significantly higher than that in the FABP group mice (*P* < 0.05). In contrast, no significant differences in Tregs frequency were observed between the infected and combination groups.

### Cytokine Production by Splenocytes

**Figure 2:**
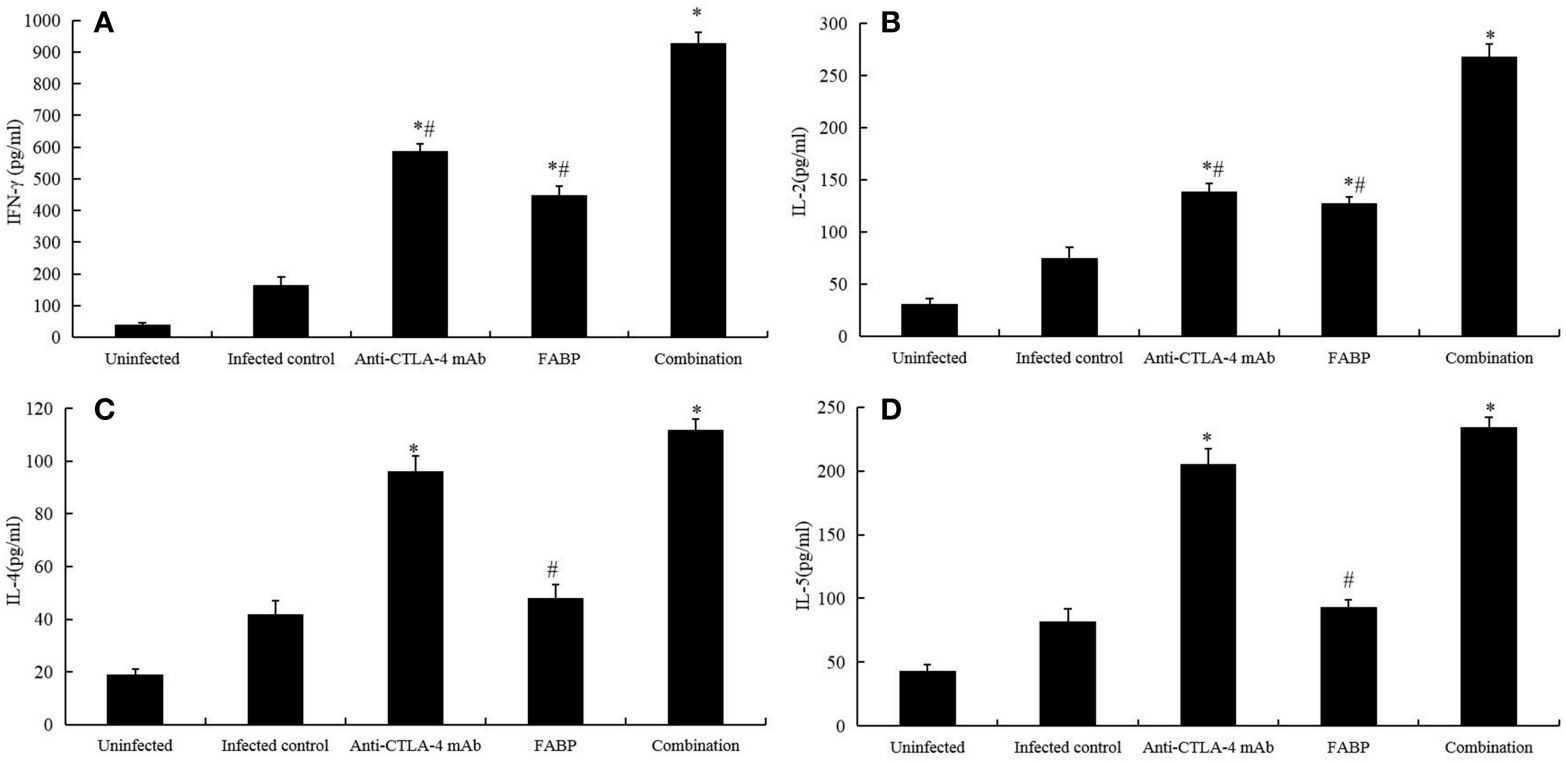
Cytokine levels in the various mouse treatment groups. The levels of the cytokines interferon (IFN)-γ **(A)**, interleukin (IL)-2 **(B)**, IL-4 **(C)**, and IL-5 **(D)** were measured in cultured spleen cells at 5 weeks post-infection with *S. japonicum*. The data are presented as the mean ± S.D of experiments performed in triplicate. *P < 0.05 compared to the infected-control group. *#P* < 0.05 compared to the combination group.

Previous studies have shown that vaccine-induced immune responses target schistosomula-stage trematodes, which normally develop into adult worms after ~4 weeks (21). In the present study, we observed the cytokine levels in 5 weeks after infection. Compared with those in the infected group, the levels of Th1 cytokines IFN-γ and IL-2 (Figures 2A,B) were higher following administration with anti-CTLA-4 mAb, FABP alone, which further elevation in the combined group. Furthermore, the levels of the Th2 cytokines IL-4 (Figure 2C) and IL-5 (Figure 2D) were increased in anti-CTLA-4 mAb and the combined groups. In contrast, no significant differences were observed between the infected and FABP groups.

### Histopathological Evaluation

**Figure 3:**
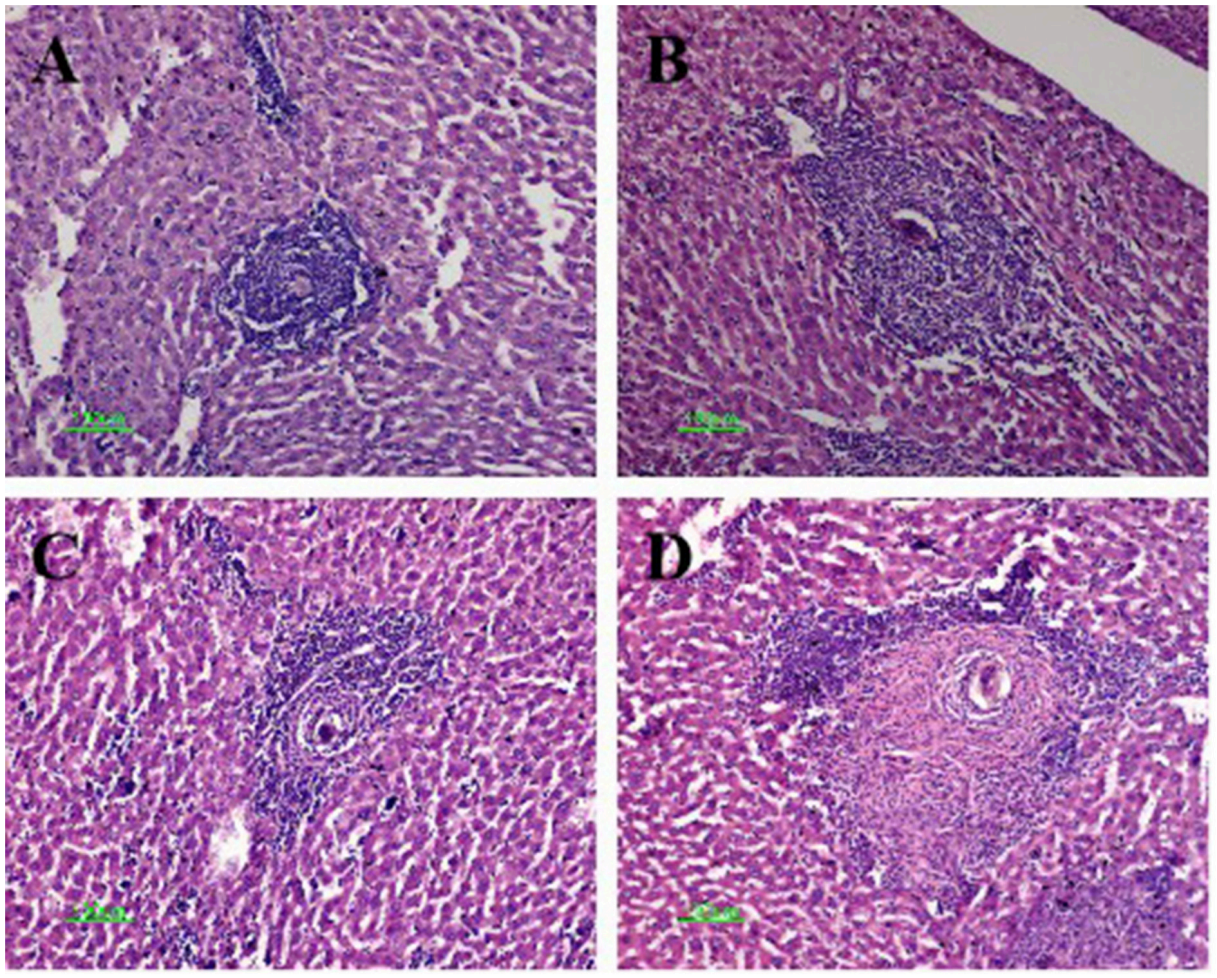
Hematoxylin and eosin staining of *Schistosoma* egg granulomas in the livers of mice in the four infected groups. Representative photos are shown for mice in the infected-control **(A)**, anti-cytotoxic T-lymphocyte antigen-4 monoclonal antibody (anti-CTLA-4 mAb) **(B)**, fatty acid-binding protein (FABP) **(C)**, and combination group **(D)**. Scale bar, 100 μm.

The mice in the four infected groups exhibited typical egg granulomas, as well as cellular infiltration including that of lymphocytes, mononuclear cells, and eosinophil granulocytes (18). Notably, the average granuloma sizes within the anti-CTLA-4 mAb-treated (306 ± 52 μm) and combination-treated (352 ± 68 μm) groups were significantly larger than those in the infected control group (152 ± 37 μm) (*P* < 0.05). The size of the granuloma was also increased in the FABP (180 ± 45 μm) compared to the infected control group; however, this increase was not statistically significant. Finally, the size of the granulomas in the combination group was significantly larger than that in the FABP group (*P* < 0.05) (Figure 3).

## Discussion

Some helminth parasitic infections stimulate the recruitment of CTLA-4^+^ T cells, and thereby escape host immune attack. For example, a previous study showed that the number of cells expressing CTLA-4 was significantly increased in mice at 1 and 2 weeks after infection with *Trichinella spiralis* (as compared to controls) (22). Similarly, Walsh et al. (23) previously identified a marked expansion of the CTLA-4^+^ population during *S. mansoni* infection. Treg CTLA-4 expression has also been shown to be upregulated in patients infected with *Plasmodium vivax*, and moreover, the level of CD4^+^CD25^+^Foxp3^+^CTLA-4^+^ T cells after *P. vivax* infection has been shown to be proportional to the host parasite load (24). Accordingly, abrogating CTLA-4 production has been demonstrated to promote parasite elimination by the host immune system. For example, administration of the anti-CTLA-4 mAb was previously reported to enhance T-cell responses, and thereby eliminate infection by the virulent bacteria, *Listeria monocytogenes* (25). Accordingly, the present study evaluated the effect of abrogating CTLA-4 function on the efficacy of the FABP vaccine against murine *S. japonicum* infection. The results of the conducted analyses showed that the efficacy of the FABP vaccine in reducing the worm burden in infected mice was increased from 26.58% (FABP group) to 54.61% (combination group) when administered in conjunction with the anti-CTLA-4 mAb. These results suggest that the anti-CTLA-4 mAb can enhance the protective effect of FABP vaccine. Consistent with this finding, Fahmy et al. (26) previously reported it to enhance the immune activity stimulated by the Bacille-Calmette-Guerin vaccine in bladder submucosal tissue.

Tregs have been shown to be interfering with, and thereby reducing the immune protective effects elicited by potential vaccines such as *S. japonicum* glutathione-S-transferase (*Sj*GST) (8). Consistent with this, the present study demonstrated a decrease in the Treg percentage in the FABP group that likely either facilitated or was associated with the protective effect exerted by the FABP vaccine. Conversely, epidermal FABP-deficient mice have been previously shown to exhibit increased levels of Tregs, and furthermore, immature CD4+ T cells isolated from these mice display enhanced Foxp3 expression (27). CTLA-4 is known to be constitutively expressed by Tregs, and is considered to promote immune suppression by enhancing their immune-inhibitory function; thus, administration of the anti-CTLA-4 mAb has been demonstrated to effectively treat various tumor types by abrogating Treg activity (28). Interestingly, the results of the present study showed that the Treg percentage was elevated in the anti-CTLA-4 mAb group, which suggests that the anti-CTLA-4 mAb enhanced the immune protective effects elicited by the FABP vaccine via an alternative mechanism, such as enhancing effector T-cell proliferation (29). In fact, CTLA-4 has been shown to promote T-cell activity, and to block the “stop” signal induced by the T-cell receptor (30); thus, inhibiting CTLA-4 is expected to increase the proliferation of various cells including Tregs. However, the interaction between the Tregs and CTLA-4 require further study. Recent studies have similarly demonstrated that the anti-CTLA-4 mAb does not reduce Tregs in bladder cancer, prostate cancer, nor melanoma (31). Moreover, a previous study showed that during filarial parasitic infection, protective immunity is prevented by a combination of increased Treg activity and CTLA-4 inhibition *in vivo* (32).

The Th1-type immune response is known to be critical for the host-mediated elimination of *S. japonicum* (33). Consistent with this, the data presented herein showed that anti-CTLA-4 mAb administration caused an increase in the levels of the Th1 cytokines IFN-γ and IL-2, strongly suggesting that anti-CTLA-4 mAb promotes *S. japonicum* elimination by enhancing the Th1-type immune response. Notably, anti-CTLA-4 mAb administration also induced an increase in the Th2 cytokines IL-4 and IL-5, supporting both that Tregs mediate schistosomiasis pathogenesis, and that CTLA-4 regulates the Th2 response to worm infection (23). Tregs contribute to the escape of *S. japonicum* from the host immune responses, while anti-CD25 mAb can partially block Tregs and thus decrease the worm and egg burden (34). In fact, the pathology caused by *S. japonicum* egg infection is considered to be a Th2-type immune response; accordingly, the granuloma diameter was shown in the present study to be significantly increased in the anti-CTLA-4 mAb and combination compared to the infected-control group. Supporting this finding, a previous study showed that CTLA-4 activity inhibits T cell-mediated parasite tissue sequestration in a mouse model of malaria, and thereby limits malaria-induced immune pathology (35). Together, these findings support that the anti-CTLA-4 mAb enhances host immune-mediated suppression of tumorigenesis and pathological reactions. Consistent with this hypothesis, a previous study used a similar schistosomiasis model to that employed here to demonstrate that the anti-CTLA-4 mAb facilitates host-mediated elimination of *S. japonicum*, but incurs an increased level of pathological damage (36). Furthermore, the anti-CTLA-4 mAb has been previously reported to exert marked therapeutic effects against various cancers and worm infections, but causes obvious body damage, i.e., immune-related adverse events, including colitis, hepatitis, and pneumonitis (37). Therefore, clinicians should carefully balance the efficacy of anti-CTLA-4 mAb treatment against incurred pathological reactions (38).

In conclusion, we herein demonstrated that the anti-CTLA-4 mAb may synergistically enhance the protective efficacy of the FABP vaccine against *S. japonicum* infection by promoting Th1 and Th2 type immune responses, while also causing increased body damage.

## Ethics Statement

All experiments in the present study were conducted with the approval of the Animal Research Committee of Wuchang Hospital (No. 2017-0027).

## Author Contributions

CT and QP were mainly responsible for literature collection and paper writing. Y-pX and YX were responsible for the experimental operation. RZ and JH are the corresponding authors, and directed the study.

### Conflict of Interest Statement

The authors declare that the research was conducted in the absence of any commercial or financial relationships that could be construed as a potential conflict of interest.
